# Effect of YAl_2_ Particles on the Corrosion Behavior of Mg–Li Matrix Composite in NaCl Solution

**DOI:** 10.3390/ma12030549

**Published:** 2019-02-12

**Authors:** Zihan Chen, Chonggao Bao, Guoqing Wu, Yongxin Jian, Li Zhang

**Affiliations:** 1 State Key Laboratory for Mechanical Behavior of Materials, School of Materials Science and Engineering, Xi’an Jiaotong University, Xi’an 710049, Shaanxi Province, China; doyen_chen@stu.xjtu.edu.cn (Z.C.); yxjian@xjtu.edu.cn (Y.J.); lily3113004001@stu.xjtu.edu.cn (L.Z.); 2 School of Materials Science and Engineering, Beihang University, Beijing 100191, China; guoqingwu@buaa.edu.cn

**Keywords:** magnesium, intermetallics, metal matrix composites, XPS, polarization, EIS

## Abstract

The strength of Mg–Li alloy is greatly improved by the composite strengthening of intermetallic compound YAl_2_ particles, but the low corrosion resistance of Mg–Li alloy is still the main factor that restricts the application of the alloy and its composites. In this paper, the effect of YAl_2_ particles on the corrosion behavior of Mg–Li alloy was systematically investigated. The results showed that the corrosion resistance of YAl_2p_/LA143 composite could be significantly improved, accounting for the formation of a transitional interface layer by adding YAl_2_ particles. The diffusion of yttrium and aluminum atoms from YAl_2_ particulates improved the stability of the surface film and enhanced the adhesion between the corrosion products and the substrate, which hindered further expansion of pitting.

## 1. Introduction

Mg–Li-based alloys are currently the lightest among the metallic structural materials, characterized with many excellent properties such as high specific strength, mechanical casting properties, good damping capacity, good thermal conductivity and electromagnetic shielding performance [1,2]. Thus, Mg–Li-based alloys have great potential to be applied in aerospace applications, automobiles, electronic products, etc. [3,4,5,6]. However, Mg–Li alloys have the inherent problems of low absolute strength, high chemical activity and poor corrosion resistance, which could decrease their mechanical stability and limit their extensive use [7,8,9,10]. It is known that compound reinforcement is a possible way to increase strength and stiffness of Mg–Li-based alloys and prevent mechanical properties degradation.

S.J.Wang and G.Q.Wu et al. [11] developed an Mg–Li matrix composite reinforced with intermetallic compounds (5 wt.% YAl_2_ particulates) by stirring casting technique. The YAl_2_ particulates uniformly dispersed inside the matrix alloy, and the mechanical properties of the composite were significantly improved. The tensile strength, elastic modulus and hardness of composite were increased by 45.3%, 44.7% and 58.2%, respectively, compared with the initial matrix alloy. In spite of the excellent mechanical properties, poor corrosion resistance has become a critical issue that restricts the applications of Mg–Li composite material. The weak corrosion resistance of Mg–Li-based alloys is mainly caused by the presence of Li, a highly electrochemical and chemically active element. The addition of Li causes a significant increase in the chemical activity of the alloy [12]. Besides, for magnesium-based composites, the addition of the enhanced phase usually exacerbates the corrosion of the matrix [13]. The structural defects caused during composite preparation, such as tiny cracks, pores, and corrosion galvanic pairs, result in increased localized corrosion of the substrate, hence the magnesium matrix composite material is generally easier to be corroded than the magnesium alloy. In addition, the enhanced phase may destroy the integrity of the protective film on the composite surface, promoting the local corrosion of the composite material. Some magnesium-based composites are severely corroded during manufacture, storage, and transportation [14]. 

In recent years, several researchers [15,16,17,18] have reported that the corrosion behavior and morphology of magnesium alloys are related to alloy composition and environmental conditions. The atmospheric corrosion mechanism of magnesium alloys is mainly caused by the electrochemical destruction of the combined action of oxygen, moisture, and corrosive media contained in the marine atmosphere. When there is a hygroscopic solid depositing on the metal surface, it can absorb water from the atmosphere and promote the formation of a thin liquid film on the metal surface, inducing and aggravating the electrochemical corrosion process. NaCl has strong hygroscopicity as one of the major solid deposits in the marine atmosphere. In addition, Cl^−^ will increase the conductivity of the electrolyte membrane and promote the dissolution of the metal. They have found that pitting corrosion tends to occur on the surface of the magnesium alloys in the presence of Cl^−^. 

Yet, to the best of our knowledge, only few researchers have reported the corrosion performance of Mg–Li alloys [19,20], as well as the systemic investigations of the corrosion behaviors. Furthermore, only few literature is available on the effect of the composite phase on corrosion performance of Mg–Li-based alloys. Therefore, the corrosion behaviors of Mg–Li matrix composite with and without YAl_2_ particles were systematically investigated by immersion test in 3.5 wt.% NaCl solution.

## 2. Materials and Methods 

### 2.1. Materials and Solutions

The YAl_2_ reinforced Mg–Li matrix composite was prepared by stirring casting in an electrical resistance furnace under a protective argon atmosphere. The raw materials included 5 wt.% YAl_2_ intermetallic compound (0.5–3 μm) and the base matrix alloy Mg-14Li-3Al (LA143). Before solidification, the superheated slurry (680 °C) was stirred at 700 r.p.m. for 30 min. After casting, the materials were extruded under 300 °C at a speed of 0.5 mm/s. The density of YAl_2p_/LA143 composite and LA143 alloy is 1.42 g/cm^3^ and 1.37 g/cm^3^.

**Figure 1:**
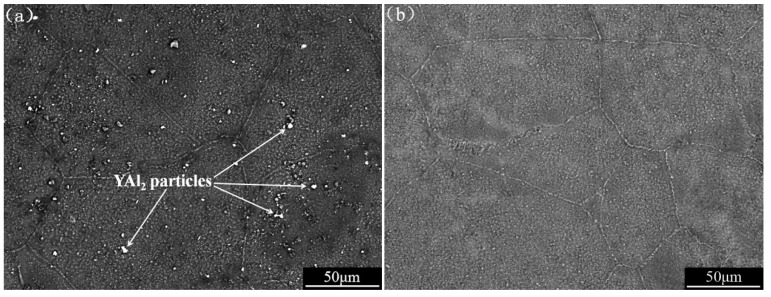
The SEM (backscatter electron) micrograph of (**a**) YAl_2_ reinforced LA143 matrix composite and (**b**) LA143 alloy.

The YAl_2_ reinforced Mg–Li matrix composite is mainly composed of YAl_2_ particles and (β)-Li phase. As shown in Figure 1a, YAl_2_ particles are uniformly dispersed in the matrix. However, the LA143 alloy only contains (β)-Li single-phase, as shown in Figure 1b. 

In the experiment, the YAl_2p_/LA143 composite and LA143 alloy were cut into individual samples with a size of Φ16 mm × 3 mm. Then the samples were orderly ground by 600#, 800#, 1000#, 1500#, 2000# SiC sand paper, and then polished on flannelette with that aiming to Ra ≤ 1.0 μm. During the polishing process, anhydrous ethanol was used to scrub the sample surface. After polishing, the sample was ultrasonically washed in acetone solution for less than 5 min.

In the marine atmosphere, magnesium alloys are vulnerable to corrosion due to the existence of Cl^−^. To simulate the corrosion medium, 3.5 wt.% NaCl solution was prepared by analytically pure reagents and distilled water during the corrosion performance test.

### 2.2. Immersion Test

Firstly, the standard samples of LA143 alloy and YAl_2p_/LA143 composite were divided into 6 groups which were then immersed into 3.5 wt.% NaCl solution at the temperature of 298 K. The testing time for each group was respectively set as 12 h, 24 h, 36 h, 48 h, 60 h and 72 h. The ratio of solution volume to specimen surface area (2 cm^2^) was 20 mL/cm^2^ according to the Chinese national standards (JB/T 7901-1999). After immersion, the corrosion products of each sample were removed by a chromic acid solution composed of CrO_3_ (200 g/L) and AgNO_3_ (10 g/L) according to the Chinese national standards (GB/T 16545-1996). The corrosion rate R (gm^−2^h^−2^) can be calculated by measuring the sample mass before and after immersion following the Equation (1):

(1) $$R=\frac{{M-M}_{1}}{\mathrm{ST}}$$

where M (g) and M_1_ (g) are the mass before and after corrosion, S (m^2^) is the area of corroded surface and T (h) is corrosion duration [21].

Meanwhile, to keep the reproducibility of the result, the same measurement were conducted three times for each group. The final data was derived from the average of the three independent measurements results.

### 2.3. Electrochemical Measurements

The corrosion resistance of LA143 alloy and YAl_2p_/LA143 composite were analyzed by ELDY/CS-310 electrochemical station (Zhonghui Tiancheng Technology Co., Ltd., Beijing, China). A three-electrode system worked during the electrochemical measurement. A platinum electrode was used as the auxiliary electrode, a saturated calomel electrode (SCE) as the reference electrode, while a standard sample with the exposed surface of 1 cm^2^ as the working electrode.

The detection time of the open circuit potential (OCP) was 1 h. The potentiometric polarization curves were used to study the protective performance of YAl_2_ metal compounds at a scan rate of 2 mV/s. Electrochemical impedance spectroscopy (EIS) was used to analyze the corrosion behavior with scanning frequencies from 10 mHz to 100 kHz. Zsimpwin software (Version 3.10, EChem Software, Ann Arbor, MI, USA) was used to collect, fit, and analyze experimental data.

### 2.4. Microstructure Characterization

The surface morphology after corrosion was observed by a scanning electron microscopy (SEM, VEGAL XMUINCA) (TESCAN, Brno, Czechoslovakia) with energy dispersive analysis of X-ray (EDAX) (Oxford Instruments, Oxford, United Kingdom). In addition, a 3D laser scanning microscope (VK-9710K) (KEYENCE, Osaka, Japan) was used to help analyze the three-dimension morphology of the corrosion surface. Corrosion products were analyzed using the X-ray diffraction (XR, X’PERT PR) (BRUKER, Karlsruhe, Germany) and X-ray photoelectron spectroscopy (XPS) (SHMADZU, Kyoto, Japan). 

## 3. Results and Discussion

### 3.1. Immersion Test

**Figure 2:**
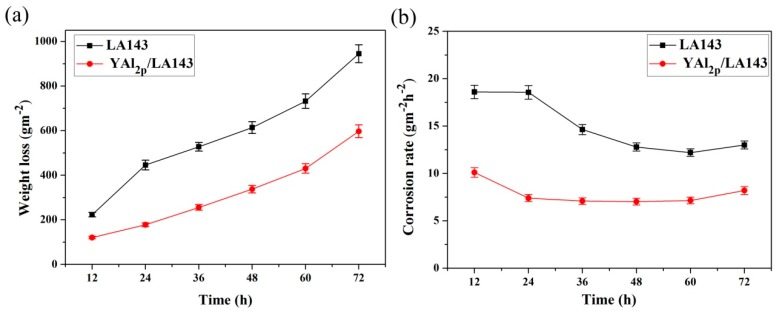
Curves of the LA143 alloy and YAl_2p_/LA143composite corrosion at 298 K for 72 h in 3.5 wt.% NaCl solution. (**a**) Weight loss versus time and (**b**) corrosion rate versus time.

Figure 2a shows the weight loss of LA143 alloy and YAl_2p_/LA143 composite after immersion in 3.5 wt.% NaCl solution as a function of time. It can be seen that the weight loss of the LA143 alloy is nearly two times larger than that of the composite. After 72 h, the weight loss of LA143 alloy rises up to 944.95 g/m^2^ while the YAl_2p_/LA143 composite is 596.88 g/m^2^. In order to further explore the corrosion process of these two materials, the corrosion rate during the immersion test was calculated according to the results of the corrosion weight loss, as shown in Figure 2b. The corrosion rate of LA143 alloy is higher before 24 h and then declines gradually. However, for YAl_2p_/LA143 composite, the corrosion rate decreases slightly before 24 h and then becomes stable. It is worth noting that the corrosion rate of both materials shows a slightly increase in the last 12 h. By comparison, the YAl_2p_/LA143 composite presents better corrosion resistance behavior.

The main reason for the poor corrosion resistance of magnesium alloys is that Mg(OH)_2_, the main component of the surface film, is not stable in acidic, neutral, and weak alkaline solutions [17,22]. In particular, when the solution contains highly corrosive ions, such as Cl^−^ which can transform the protective MgO/Mg(OH)_2_ into soluble MgCl_2_, the dissolution of magnesium may be accelerated [23]. At the same time, a large amount of H_2_ is precipitated during the corrosion process, which further reduces the denseness of the surface film.

In the initial stage of corrosion, the corrosion rate is high because the surface film of the LA143 alloy and the YAl_2p_/LA143 composite material are not well protected. When the sample is directly exposed to NaCl solution, it is severely damaged by Cl^−^. As the duration of corrosion increases, the corrosion products are gradually formed on the surface of the material, which prevent the diffusion of the Cl^−^ to the substrate. In addition, the presence of the corrosion product layer hinders the efficient transport of charge, resulting in a decrease of corrosion rate.

From the above results, we conclude that the addition of YAl_2_ particulates plays a significant role in inhibiting the corrosion solution, and thus, improves the corrosion resistance of the composites to a certain degree. However, the mechanism of action should be comprehensively analyzed.

In order to explore the mechanism of YAl_2_ particles on the surface film and corrosion resistance of Mg–Li alloy, the surface corrosion morphologies of LA143 alloys and YAl_2p_/LA143 composites after 72 h immersion were observed and analyzed. Before we removed the corrosion products from the sample surface, we observed that the corrosion products on the composite were more cohesive than those on the LA143 alloy. The specific difference between the two samples after 72 h immersed was that the corrosion products on LA143 alloys gradually fell off as the corrosion proceeded. On the contrary, the most corrosion products on the YAl_2p_/LA143 composite were adhered to the surface of the sample, but it was too loose to protect the matrix from Cl^−^ erosion and could be removed easily. 

**Figure 3:**
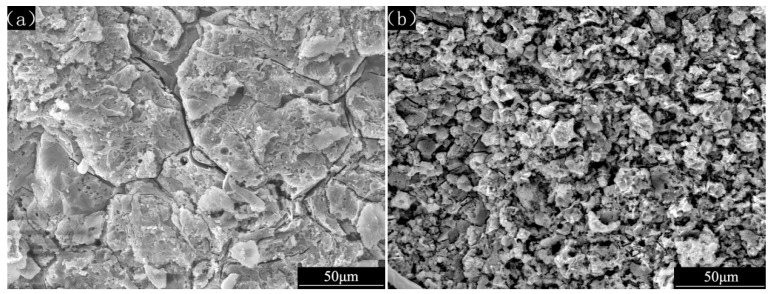
The SEM (secondary electron) micrograph of (**a**) LA143 alloys and (**b**) YAl_2p_/LA143 composites’ surface after 72 h immersion in 3.5 wt.% NaCl solution.

**Figure 4:**
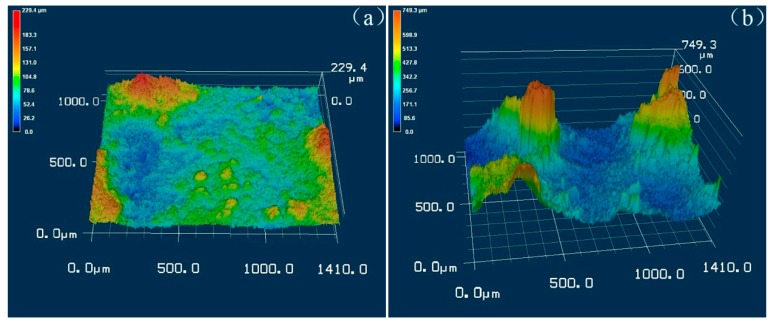
The 3D laser scan image of (**a**) LA143 alloys and (**b**) YAl_2p_/LA143 composites’ surface after 72 h immersion in 3.5 wt.% NaCl solution.

Figure 3 shows the surface corrosion morphologies of LA143 alloys and YAl_2p_/LA143 composites after 72 h immersion in 3.5 wt.% NaCl solution after removing the corrosion products. It can be seen that the corrosion surface of the LA143 alloys (Figure 3a) is flatter than the YAl_2p_/LA143 composites (Figure 3b), indicating the uniformity corrosion of the LA143 alloy. On the contrary, the corroded surface of the composite is very rough, with a large number of crater-shaped protrusions and numerous corrosion pits. Furthermore, the three-dimensional morphologies of the corrosion surface were detected by 3D laser scanning microscopy, as shown in Figure 4. Compared with the LA143 alloy, several protrusions can be obviously observed on the corrosion surface of the YAl_2p_/LA143 composites, which is consistent with the SEM observations. These protrusions are supposed to be the YAl_2_ particles. In this case, the protruded YAl_2_ particles may change the corrosion mode and hinder the further expansion of the pitting, which benefits to improvement of the corrosion resistance.

**Figure 5:**
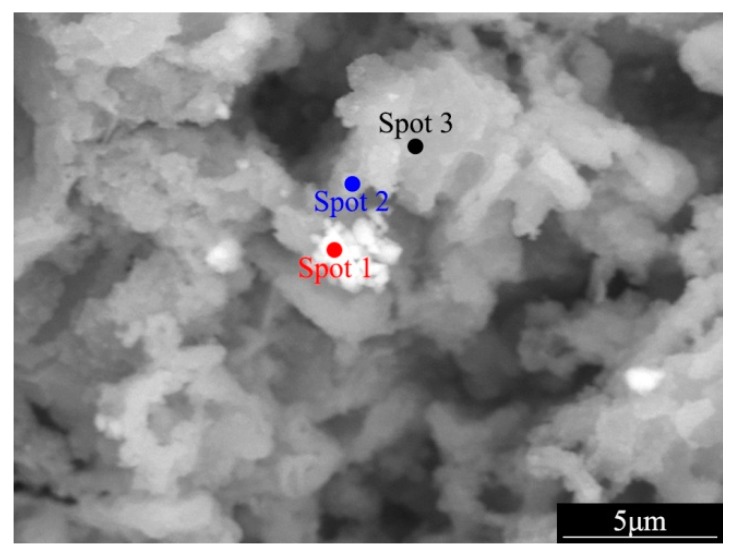
The EDS image (backscatter electron) of YAl_2p_/LA143 composites’ surface after 72 h immersion in 3.5 wt.% NaCl solution.

In order to clarify the formation of the characterized corrosion surface of the YAl_2p_/LA143 composite, the chemical composition of the protrusion was determined by EDS (Energy Dispersive Spectrometer), as shown in Figure 5. In this figure, three spots were detected on the top of the protrusions. Spot 1 is exactly on the YAl_2_ particle, while spot 2 and spot 3 are gradually away from the YAl_2_ particle on the base alloy. The detecting results of the chemical compositions are shown in Table 1. The weight percentages of Al (15.76%) and Y (28.16%) are relatively high, as the detecting point is on the YAl_2_ particle. It is worth noting that Al (7.24% and 7.44%) and Y (7.96% and 7.43%) could also be detected on spots 2 and 3. In this context, it can be concluded that Al and Y could diffuse into the surrounding substrate. In the previous study [24], due to the agglomeration problem within the matrix caused by the size reduction of the reinforcement particles during the composite preparation, the YAl_2_ particles were surface-modified with a compound ball mill method. During ball milling, the surface of YAl_2_ particles was covered by Mg film. With the extension of ball milling time and the continuous loading of mechanical force, atomic diffusion occurred between YAl_2_ and Mg, forming a metallurgical bonding interface. Therefore, in the composite material prepared by adding this YAl_2_/Mg composite powder to the matrix alloy, there exists a transitional interface layer with a certain width between the reinforcement and the base body, which is dominated by the diffusion of yttrium and aluminum atoms.

Several researchers found that the addition of Al and Y into certain commercial alloys (AZ91) or binary Mg alloys (Mg–Y, Mg–Al) had a positive effect on the corrosion resistance [25,26,27]. On the one hand, in the neutral environment, Al can form Al_2_O_3_ phase to protect the matrix on the surface of magnesium alloy. Additionally, Al addition can also improve the stability of the Mg(OH)_2_ film, benefiting to the improvement of the corrosion resistance. On the other hand, Y has the same standard electrochemical potential (−2.372 V SHE (Standard Hydrogen Electrode)) as Mg, which can tremendously improve the corrosion resistance of the magnesium alloy [27]. Luo et al. [25] reported that the corrosion resistance of AZ91 alloy could be improved with Y addition less than the critical content of 0.3 wt.%. Therefore, the diffusion of Y and Al is supposed to improve the corrosion resistance of the YAl_2p_/LA143 composite. 

**Figure 6:**
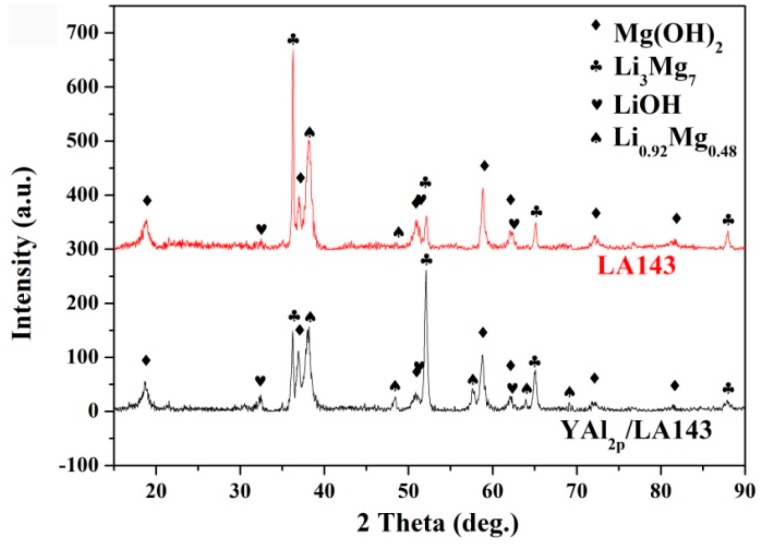
XRD analysis of LA143 Alloy and YAl_2p_/LA143 composite, after immersion test in 3.5 wt.% NaCl solution.

**Figure 7:**
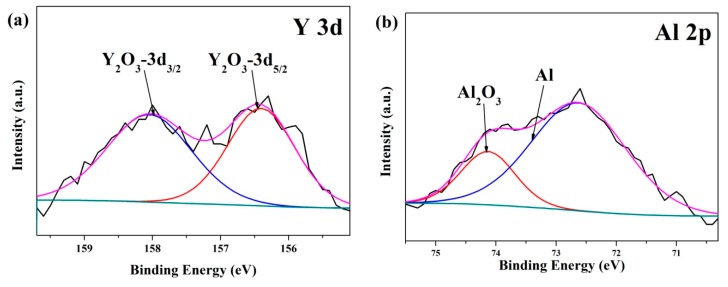

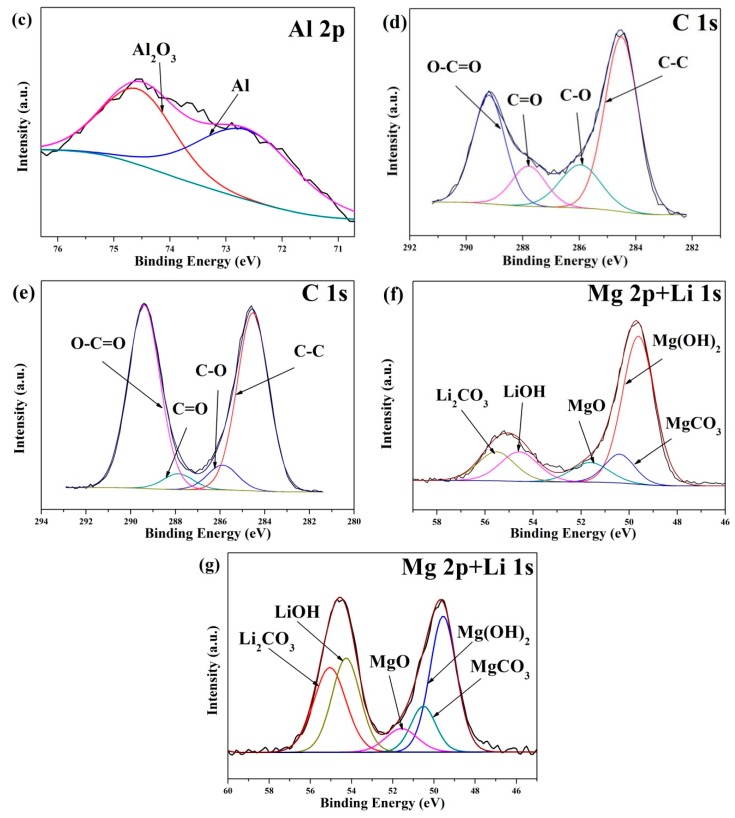
XPS analysis of (**b**,**d**,**f**) LA143 Alloy, and (**a**,**c**,**e**,**g**) YAl_2p_/LA143 composite, after immersion test in 3.5 wt.% NaCl solution.

In order to investigate the effect of diffused Y and Al on the surface film of the YAl_2p_/LA143 composite, the corrosion products of the alloy and the composite were analyzed by XRD and XPS. The XRD spectrum of the corrosion products are shown in Figure 6 that the corrosion products of both alloy and composite are Mg(OH)_2_ and LiOH. In addition, an incomplete corrosion phase of Li_0.92_Mg_0.48_ is detected, the appearance of which can reflect the corrosion has been suppressed to a certain extent. Compared with the LA143 alloy, the corrosion products of YAl_2p_/LA143 composite contains higher content of Li_0.92_Mg_0.48_, indicating the composite exhibits a higher degree of corrosion inhibition. The XPS analysis suggests that the compositions of the immersion product film on the surface of both LA143 alloy and YAl_2p_/LA143 composite are mainly composed of Mg(OH)_2_, LiOH, MgCO_3_, Li_2_CO_3_ and a small amount of Al_2_O_3_, as shown in Figure 7. However, Y_2_O_3_ (Figure 7a) can be found in YAl_2p_/LA143 composite which is formed by the diffusion of Y from YAl_2_ particles. According to the reports of Luo et al. [25], Y_2_O_3_ can help to improve the stability of the surface film. From Figure 7b,c, it is shown that the diffusion of Y promotes the transition from Al to Al_2_O_3_, which improves the relative density of the surface film. As shown in Figure 7d–g, a significant increase of Li_2_CO_3_ is observed according to the peaks of C 1s and Li 1s, which agrees well with the results of Eriksson et al. [28]. On the other hand, Xu et al. [29] found, Mg and Li would oxidize preferentially to produce MgO and Li_2_O when the Mg–Li alloy was exposed to atmospheric air. Subsequently, Li_2_O reacted with atmospheric CO_2_ to generate the Li_2_CO_3_ layer. Due to the existence of Y, Li_2_O can actively react with CO_2_ to generate more Li_2_CO_3_. The possible reaction process is as the following Equations (2) and (3):

4Li + O_2_ = 2Li_2_O (2)

Li_2_O + CO_2_ = Li_2_CO_3_ (3)

The presence of Li_2_CO_3_ is greatly beneficial to the matrix alloy because of its indissolubility in water and limits the dissolution of the alloy. In Figure 6f and g, the peak of LiOH in YAl_2p_/LA143 composite is obviously higher than that in LA143 alloy. Since LiOH tends to dissolve into the solution, the Li_2_CO_3_ layer in the product film can help hinder the dissolution process of LiOH. In YAl_2p_/LA143 composite, the product film is relatively denser, resulting in the better protective effect. 

### 3.2. Open Circuit Potential and Potentiodynamic Polarization Measurements

**Figure 8:**
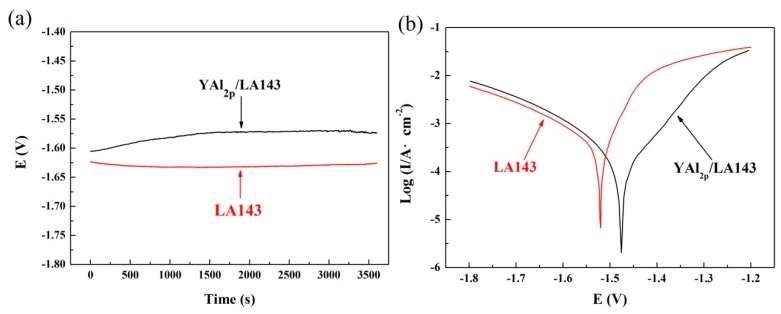
The curves of (**a**) open circuit potential vs time and (**b**) polarization of YAl_2p_/LA143 composite and LA143 alloy in 3.5 wt.% NaCl solution.

The curves of open circuit potential versus time of YAl_2p_/LA143 composite and LA143 alloy in 3.5 wt.% NaCl solution for 1 h are shown in Figure 8a. As we can see, the OCP of the composite is around −1.578 V, and the OCP of LA143 alloy is about −1.623 V. Although OCP is not related to the corrosion rate, it is still able to reflect the formation process and chemical stability of the oxidation film on the sample. For LA143 alloy, the OCP declines slowly at the beginning and then becomes stable. However, the change in the OCP of the composite, contrary to that of the alloy, experiences a slow rising at the beginning before its stabilization thereafter. Finally, the potential difference between the alloy and composite is about 0.05 V. This demonstrates that during the corrosion process, the film formed on the surface of the composite is more protective than the alloy. From another perspective, when the composite coupled to other materials, it has a lower galvanic corrosion tendency.

The polarization curves of YAl_2p_/LA143 composite and LA143 alloy in 3.5 wt.% NaCl solution are shown in Figure 8b. The cathodic polarization curves represent the cathode hydrogen evolution through water reduction, whereas the anodic polarization curves show the active dissolving of Mg–Li alloy. As shown in Figure 8b, the YAl_2_ particles have little effects on cathodic polarization process. But, the anodic part of YAl_2p_/LA143 composite is characterized by a slowly increase of current density with increasing potentials. However, for LA143 alloy, the current density increases sharply once exceeding the corrosion potential. The lower dissolution rate of YAl_2p_/LA143 composite reveals that a compact and protective corrosion product film is formed on the surface of YAl2p/LA143 composite, which is consistent with the results of XPS analysis (Figure 7). In addition, the corrosion potential of YAl_2p_/LA143 (-1.476V) composite is more positive than that of LA143 alloy (−1.521 V). 

Generally, the Magnesium alloys often behave as ideal non-polarizable electrodes. Moreover, the anodic branch is strongly affected by the well-known negative difference effect. Therefore, the anodic “Tafel” slope does not correspond to the activation of the charge transfer process. In this case, the corrosion current density was determined only for the estimation of the cathodic currents using the well measured cathodic branch. The corrosion current density (Icorr) of YAl_2p_/LA143 composite and LA143 alloy is 6.6674 × 10^−4^ and 8.6097 × 10^−4^. Despite their corrosion current density are in the same order of magnitude, the composite still shows a better corrosion resistance.

### 3.3. EIS Characteristics

**Figure 9:**
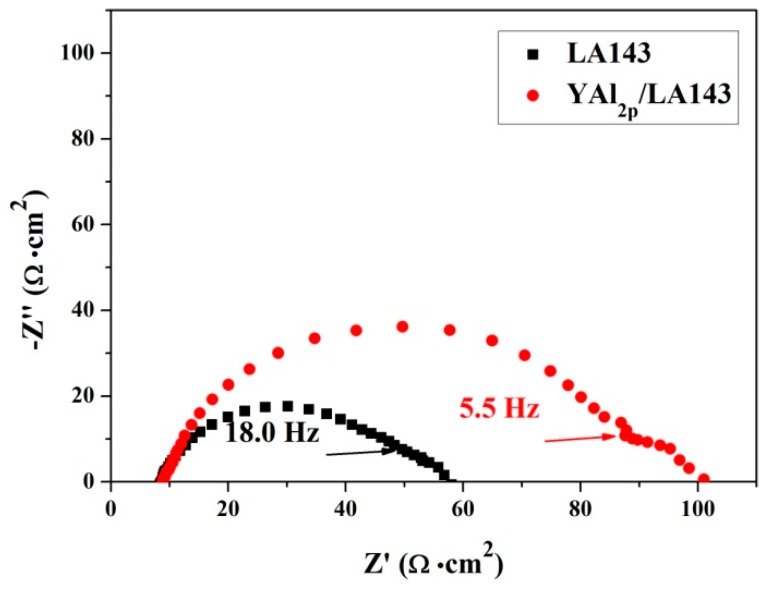
Nyquist plots of LA143 and YAl_2p_/LA143 in 3.5% NaCl solution.

**Figure 10:**
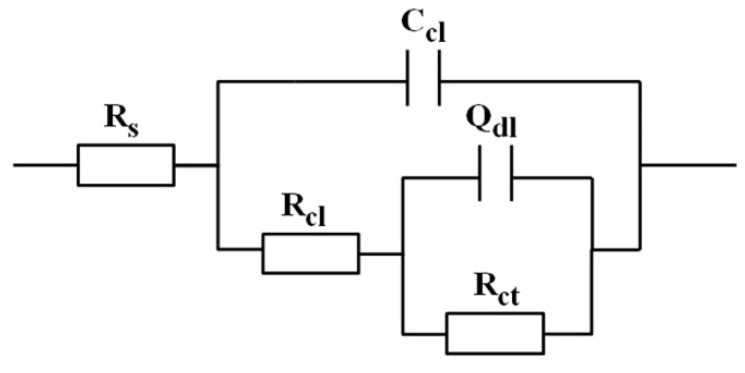
Electrical equivalent circuit diagrams to fit the impedance spectra of LA143 and YAl_2p_/LA143 in 3.5% NaCl solution in Figure 9. R_s_: solution resistance; C_cl_: corrosion layer capacitance; R_cl_: corrosion layer resistance; Q_dl_: double-layer capacitance, R_ct_: charge transfer resistance.

Figure 9 presents the Nyquist plots of YAl_2p_/LA143 composite and LA143 alloy in 3.5 wt.% NaCl solution. The plot of each sample consists of two capacitive loops. The high-medium frequency capacitive loop is probably attributed to the mass transport resistance of surface film, while the low frequency capacitive loop is related to the electric double layer capacitance and charge transfer resistance [30]. EIS equivalent circuit is proposed to model the sample/solution interface of YAl_2p_/LA143 composite and LA143 alloy in 3.5 wt.% NaCl solution as shown in Figure 10. The values of equivalent circuit component are summarized in Table 2 after fitting process. 

As we can see, the capacitive loop of composite is twice as big as that of the alloy. In general, a large capacitive loop means good corrosion resistance and a low corrosion rate. Besides, the value of R_cl_ and R_ct_ in the equivalent circuit of LA143 alloy is lower than that of the composite, while the value of C_cl_ and Q_dl_ is higher. The value of C_cl_ can represent the density of corrosion layer. The value is big when the layer is loose textured [31,32,33,34,35,36,37]. It is concluded that the addition of YAl_2_ particles improves the density of corrosion product. The resistance of corrosion product increases, while the capacitance decreases, which means the YAl_2p_/LA143 composite has a better corrosion resistant property.

As above immersion test and electrochemical test results mentioned, the addition of YAl_2_ particles not only greatly improves the mechanical properties of the composite material, but also suppresses the corrosion tendency of the matrix. Meanwhile, the diffusion of Y and Al from YAl_2_ particles improves the corrosion resistance of YAl_2p_/LA143 composite. 

## 4. Conclusions

The corrosion weight loss of the YAl_2p_/LA143 composite after being immersed in 3.5 wt.% NaCl solution for 72 h is only 63% of that of the LA143 alloy, and the corrosion rate of composite is always kept at a low level. The corrosion surface of the LA143 alloys is uniform, but the corrosion surface of the YAl_2p_/LA143 composites is very rough and possesses a large number of protrusions, and there are numerous corrosion pits. The existence of YAl_2_ contributes to the formation of a compact film on the surface of the substrate and enhances the adhesion between the corrosion products and the substrate by forming Y_2_O_3_. Furthermore, Al_2_O_3_ and Li_2_CO_3_ in the product film can be also increased, which hinders further expansion of pitting, improving the corrosion resistance of the composite material.

The addition of YAl_2_ particles makes the open circuit potential and the corrosion potential of the composite shift positively by 0.05 V, respectively. The anodic polarization curves and Nyquist plots both reveal that a compact and protective corrosion product film is formed on the surface of YAl_2p_/LA143 composite. As a result, it improves the corrosion resistance. 

## Figures and Tables

**Table 1:** The weight percentage of element for spot scanning in Figure 5.

Element	Weight Percentage (wt.%)		
	Spot 1	Spot 2	Spot 3
C	0.70	0.60	0.72
O	7.25	5.02	5.10
Mg	16.70	30.99	44.85
Al	15.76	7.24	7.44
Cl	0	1.34	0.87
Cr	31.43	46.85	33.58
Y	28.16	7.96	7.43

**Table 2:** The parameters of the equivalent circuit of LA143 and YAl_2p_/LA143 in 3.5 wt.% NaCl solution.

Samples	R_cl_/Ω·cm^−2^	C_cl_/F·cm^−2^	R_ct_/Ω·cm^−2^	Q_dl_/F·cm^−2^
LA143 alloy	27.59 ± 0.01	(4.139 ± 0.001) × 10^−5^	13.56 ± 0.01	(2.083 ± 0.008) × 10^−4^
YAl_2p_/LA143 composite	61.35 ± 0.02	(2.445 ± 0.001) × 10^−5^	17.02 ± 0.02	(1.350 ± 0.01) × 10^−4^
